# Wavelength and Polarization Multiplexed Nonlocal Metasurface for Quantitative Phase Microscopy

**DOI:** 10.1002/nap2.70190

**Published:** 2026-07-01

**Authors:** Haiwei Wang, Shikun Ma, Shaban Sulejman, Niken Priscilla, Wendy S. L. Lee, Peter Francis Mathew Elango, Lukas Wesemann, Elizabeth Hinde, Ann Roberts

**Affiliations:** ^1^ ARC Centre of Excellence for Transformative Meta‐Optical Systems, School of Physics The University of Melbourne Victoria Australia; ^2^ School of Physics The University of Melbourne Victoria Australia; ^3^ Department of Biochemistry and Pharmacology The University of Melbourne Victoria Australia; ^4^ Functional Materials and Microsystems Research Group and the Mico Nano Research Facility RMIT University Melbourne Australia; ^5^ ARC Centre of Excellence for Transformative Meta‐Optical Systems RMIT University Melbourne Australia

**Keywords:** metasurfaces, optical image processing, quantitative phase imaging

## Abstract

Imaging transparent samples remains an ongoing challenge in the study of unstained biological cells and material samples. Widely used methods trade off system complexity, cost and bulk, computational efficiency, and information content. Here, a nonlocal metasurface located in the object plane is demonstrated for obtaining single‐shot, low‐noise differential phase contrast images visualizing phase gradients along orthogonal directions in a sample obtained at wavelengths of 613 and 656 nm. These images are sufficient to calculate the quantitative phase introduced into the transmitted optical field by the sample, with experiments showing the recovered phase generated by a spatial light modulator is in good agreement with specified values. This method was also demonstrated to obtain information‐rich differential phase contrast images of unstained HeLa cells with the recovered phase excursion values consistent with the literature. These results demonstrate the potential for metasurfaces as a platform for extracting information from an optical field for use in next‐generation compact imaging systems with applications in medical diagnostics, biotechnology, and materials science.

## Graphical abstract

A nonlocal metasurface with wavelength and polarization multiplexed optical transfer functions is demonstrated for performing quantitative phase imaging. The metasurface uses the angular dispersion of optical routing guided mode resonances to extract phase gradient information along different directions for phase retrieval.

## 1 Introduction

Phase microscopy is a powerful technique where variations in the phase introduced into an optical field by a sample can be observed [1]. Due to the sensitivity of the optical phase to minute variations in the sample's refractive index and/or thickness, phase microscopy permits the observation of otherwise low‐contrast samples without staining or the introduction of fluorescent labels. As such, phase contrast microscopies have become indispensable tools in biology and materials science, with the two most popular techniques being Zernike phase contrast [2], and differential interference contrast microscopy (DIC) [3]. However, both methods have limitations. DIC visualizes phase gradients along only a single direction and Zernike phase contrast requires careful alignment and can produce artefacts. Furthermore, these techniques generate only qualitative phase images where the phase is not directly measured. Quantitative phase microscopy (QPM), on the other hand, is an imaging modality that provides a numerically evaluated phase map of the sample. Not only does QPM confer the key advantages of phase contrast microscopies, the quantitative phase data obtained can also be used in additional analyses. For example, the additional information can be used to obtain the dry mass of cells and can be used to study the growth of cells over time [4]. Existing interferometric [5, 6, 7, 8, 9, 10] and noninterferometric methods [11, 12, 13, 14, 15, 16] that permit the quantification of phase generally utilize complex and bulky systems and/or require capturing multiple images.

In the effort to miniaturize phase imaging systems, there is ongoing interest in the application of metasurfaces to phase imaging. The approaches adopted vary widely highlighting the potential of metasurfaces to underpin a variety of imaging techniques. A recent review can be found in Priscilla et al. [17]. Recently, QPM has been demonstrated predominantly using wavefront‐shaping local metasurfaces to obtain information for input into various phase recovery algorithms [18, 19, 20, 21, 22]. Inspired by differential interference contrast (DIC) microscopy, for example, the authors in ref. [18] cascaded two metasurfaces to produce three DIC images with different phase offsets permitting quantitative recovery of the phase gradient along one direction in the sample. Engay et al. [19], on the other hand, used a single metasurface located in the Fourier plane of a 4*f* system to generate two spatially separated copies of an image with defocus introduced into one to generate in a single shot images for incorporation into the transport of intensity equation. Finally, Yang et al. [23] recently demonstrated QPM and other imaging modalities with a geometric phase metasurface that obtains a measurement of the phase gradient along only one direction. An algorithm assuming weak variations along the orthogonal direction permits the determination of the quantitative phase in some cases.

In contrast to these and other local metasurfaces that introduce a point‐by‐point variation in transmission through the device, metasurfaces that perform direct spatial frequency filtering without the requirement for a 4*f* optical system or similar [24] have been demonstrated primarily for qualitative phase imaging [25, 26, 27], with only a few examples that demonstrated phase quantification experimentally [28, 29]. These metasurfaces generate wavevector‐dependent transmission (or reflection) with dispersive resonances such as guided mode resonances [28, 30, 31], resonances in multilayer film stacks [32, 33, 34], Mie resonances [35, 36], or quasi‐bound‐states‐in‐the‐continuum [37, 38]. Unlike wavefront‐shaping metasurfaces, metasurfaces capable of direct spatial frequency filtering do not have inherent imaging capabilities and can be integrated into existing imaging systems by placing them after the object or in the image plane. As is the case with local devices, the implementations are quite diverse but as an example, Ji et al. [28] demonstrated the use of a surface relief grating to produce phase contrast images and demonstrated the acquisition of quantitative phase images of binary transparent objects obtained after the calibration of their system with known samples. Most metasurfaces with the capacity to directly manipulate spatial frequencies that have been experimentally realized, including this one, exhibit a symmetric response about normal incidence making them suitable only for the extraction of edge features [36, 37, 39, 40], or for generating contrast similar to Zernike phase contrast [28] since the sign of the gradient is lost when the image is obtained, unless used with off‐normal illumination [26]. The use of an asymmetric angular transmission response about normal incidence, on the other hand, permits both the magnitude and sign of the phase gradient to be determined, providing an avenue to QPM by obtaining the gradients of the phase and subsequent processing [29, 41]. To obtain a robust quantitative phase image using this approach, however, gradient contrast along different directions within the sample is required, often leading to increased system size or complexity [14, 20, 29, 42]. Li et al. [29] demonstrated this experimentally, albeit with a pair of nonlocal thin film filters tilted about orthogonal axes and placed in a 4*f* imaging system to extract information about orthogonal phase gradients, resulting in a bulky system.

In this work, we propose and experimentally demonstrate the use of a single‐layer nonlocal guided mode resonance metasurface, multiplexing phase information in two circular polarizations and at two different wavelengths to perform QPM as illustrated in Figure 1. As part of this process, two single‐shot differential phase contrast (DPC) images visualizing phase gradients along orthogonal directions are obtained, which serve as inputs to the phase retrieval algorithm. At each of the two operating wavelengths, the sign of the gradient contrast is reversed in the left circularly polarized (LCP) and right circularly polarized (RCP) images obtained with our metasurface, which are obtained simultaneously using a polarization‐sensitive camera. These two images combine to produce a DPC image with relatively low noise, and with background contrast and other artefacts removed. By changing the wavelength, the direction of the gradient contrast is switched between the horizontal and vertical directions, permitting the acquisition of DPC along two axes for robust quantitative phase recovery. The nonlocal metasurface used here is based on the concept of optical routing into resonant waveguide gratings [27, 43, 44]. Using our platform, we demonstrate QPM of phase targets generated using a spatial light modulator (SLM) as well as of unstained HeLa cells. In contrast to previously reported work, our approach utilizes a single metasurface and does not require access to the Fourier plane, any calibration, off‐normal illumination, nor any moving parts. It produces high quality differential phase contrast images with the gradient direction wavelength multiplexed. Furthermore, the capacity to obtain phase gradient information along two orthogonal directions produces a robust and compact approach to quantitative phase microscopy. This work opens opportunities for future developments in metasurface‐based, ultracompact QPM systems that can be used in point‐of‐care and remote settings.

**FIGURE 1:**
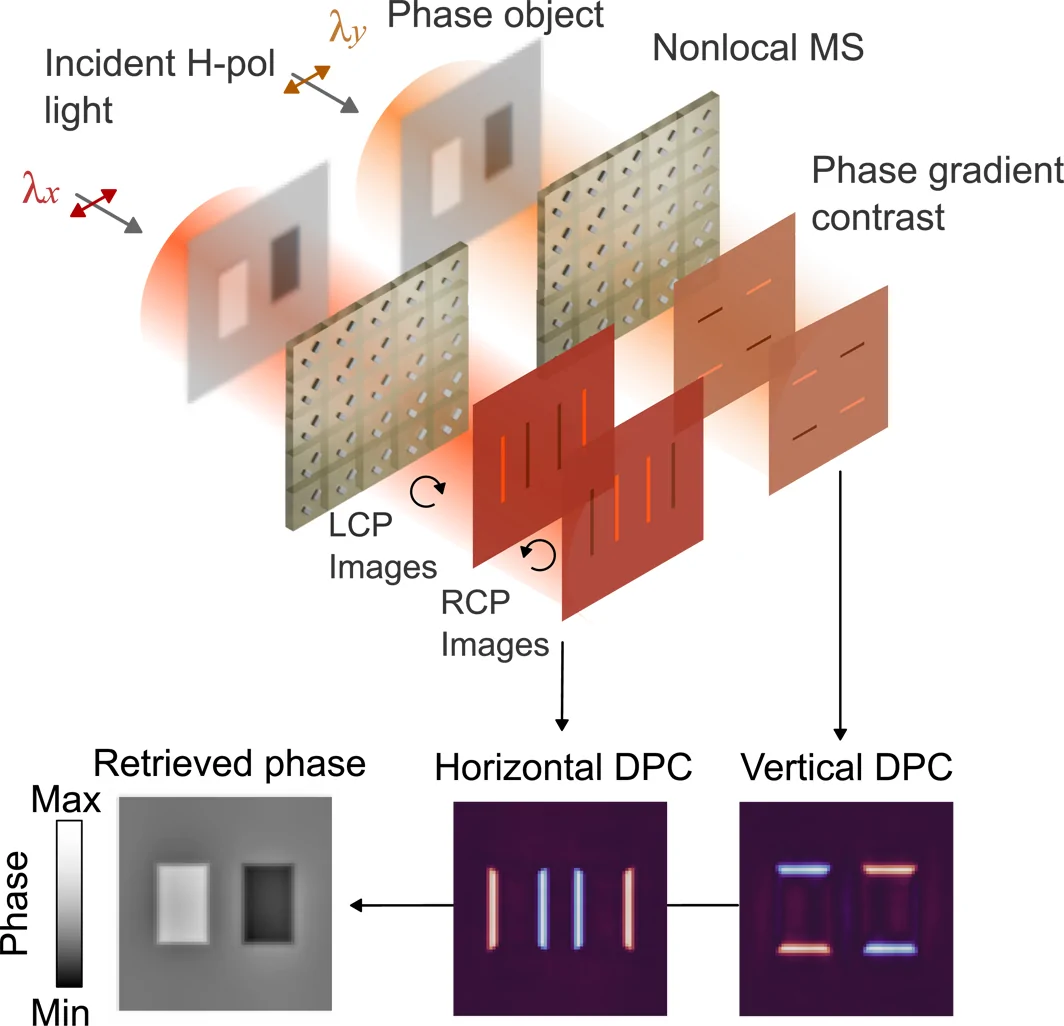
A phase object modeled as a thin, pure phase transmission mask is illuminated with horizontally polarized light at two different wavelengths. After the phase‐modulated field is transmitted through the metasurface, phase gradient contrast images with opposing contrast directions appear in the LCP and RCP channels, and the phase gradient contrast along orthogonal directions at the two wavelengths channels. The images are used to construct horizontal and vertical gradient DPC images, which are then used to retrieve the phase.

## 2 Results

### 2.1 Theoretical Framework

The objective of QPM is to determine the spatially varying phase, ϕ(ρ), a function of the transverse position ρ=(x,y), imprinted onto a transmitted field by a transparent sample. The sample is modeled as a thin, pure phase object with a transmission function:

(1) t(ρ)=exp[iϕ(ρ)].

We assume that ϕ(ρ) is polarization‐independent and is given by

(2) $$\phi \left(\rho \right)=\frac{2\pi }{\lambda }\text{OPL}\left(\rho \right),$$

where λ is the free‐space wavelength and OPL(ρ) is the optical path length at a particular point on the sample. We assume that the OPL(ρ) does not depend on wavelength such that ϕ(ρ) is inversely proportional to the wavelength, permitting the incorporation of DPC images obtained at different wavelengths into a phase retrieval algorithm [45].

The sample is illuminated by a monochromatic, normally incident plane wave propagating along the z‐axis with wavelength λ, amplitude $E_{0}$, and spatially uniform polarization given by the vector ${\hat{e}}_{\text{in}}$. To describe the effect of a nonlocal metasurface, the spatial dependence of the field is decomposed into a sum of spatial frequencies specified by coordinates $u=\left(u_{x},u_{y}\right)$, with amplitudes calculated using the Fourier transform as defined in Supporting Information S1: Supplementary Information 1. In free space, each value of u corresponds to a plane wave propagating at a distinct angle to the z‐axis and incident on the metasurface. Assuming the incident light is scattered by only small angles by the object, the polarization upon scattering remains unchanged, and the Fourier spectrum of the field incident on the metasurface can be written as $E_{\text{in}}\left(u\right)=E_{0}\tilde{t}\left(u\right){\hat{e}}_{\text{in}}$, where $\tilde{t}\left(u\right)=F\left\{t\left(\rho \right)\right\}\left(u\right)$, and F is the Fourier transform operator as defined in Supporting Information S1: Supplementary Information 1. A nonlocal metasurface with an angle dependent transmission modifies the spatial frequency spectrum of an incident field passing through it, this is characterized by a spatial frequency and wavelength dependent transmission tensor $T_{\lambda }\left(u\right)$. After transmission through the metasurface and prior to detection, the field component with polarization given by ${\hat{e}}_{\text{out}}$ is selected by means of a polarization analyzer. Finally, the measured intensity is proportional to the absolute value squared of this field:

(3) $$I\left(\rho ;\lambda ,{\hat{e}}_{\text{in}},{\hat{e}}_{\text{out}}\right)=|E_{0}{|}^{2}|F^{-1}\left\{H\left(u;\lambda ,{\hat{e}}_{\text{in}},{\hat{e}}_{\text{out}}\right)\tilde{t}\left(u\right)\right\}{|}^{2},$$

where $F^{-1}$ is the inverse Fourier transform, and $H\left(u;\lambda ,{\hat{e}}_{\text{in}},{\hat{e}}_{\text{out}}\right)={\hat{e}}_{\text{out}}\cdot T_{\lambda }\left(u\right)\cdot {\hat{e}}_{\text{in}}$ is defined as the coherent transfer function (CTF). We highlight here that different CTFs can be obtained for a metasurface with a fixed $T_{\lambda }\left(u\right)$ by switching λ and the input and output polarizations, enabling the transfer function to be selected externally without changing the metasurface or adding or removing components.

The DPC images are calculated from two intensity images with opposite gradient contrast $I_{+}\left(\rho \right)$ and $I_{-}\left(\rho \right)$:

(4) $$I_{\text{DPC}}\left(\rho \right)=\frac{I_{+}\left(\rho \right)-I_{-}\left(\rho \right)}{I_{+}\left(\rho \right)+I_{-}\left(\rho \right)}.$$

Here, these two images are obtained using a quarter waveplate in conjunction with a polarization‐sensitive camera. In this case, the advantage of using this DPC signal in phase retrieval as opposed to raw images is that it is relatively low noise and removes background information. The former is due to subtraction of the two gradient‐reversed images, where any variations common to both images (such as speckle) cancel, whereas the phase signal is reinforced. The latter benefit arises from normalizing by the sum of the two images, which removes the dependence on incident intensity, introduced, for example, by variations in absorption in the sample under investigation, permitting phase quantification without requiring a separate background (object‐free) measurement.

Following the derivation presented in Supporting Information S1: Supplementary Information 2, we show that the DPC signal depends only on the phase of the object through a convolution:

(5) $${\tilde{I}}_{\text{DPC}}\left(u\right)=H_{\text{DPC}}\left(u\right)\tilde{\phi }\left(u\right),$$

where $\tilde{\phi }\left(u\right)$ and ${\tilde{I}}_{\text{DPC}}\left(u\right)$ are, respectively, the Fourier transforms of the phase and the DPC image, whereas $H_{\text{DPC}}\left(u\right)$ is the DPC transfer function, which can be calculated from the CTFs as detailed in Supporting Information S1: Supplementary Information 2. Given a set of DPC signals as shown in Figure 1, the phase is then retrieved using noniterative deconvolution with Tikhonov regularization [46]:

(6) $$\phi \left(\rho \right)=F^{-1}\left\{\frac{\sum_{n}H_{\text{DPC},n}^{*}\left(u\right){\tilde{I}}_{\text{DPC},n}\left(u\right)}{\sum_{n}|H_{\text{DPC},n}\left(u\right){|}^{2}+\alpha }\right\},$$

where ${\tilde{I}}_{\text{DPC},n}\left(u\right)$ are the Fourier transforms of DPC signals each related to the phase via the DPC transfer function $H_{\text{DPC},n}\left(u\right)$, and α is a regularization parameter selected to balance the requirements of minimizing noise while optimizing spatial resolution. Here, two DPC images $H_{\text{DPC},x}\left(u\right)$ and $H_{\text{DPC},y}\left(u\right)$ are measured, corresponding to transfer functions $H_{\text{DPC},x}\left(u\right)$ and $H_{\text{DPC},y}\left(u\right)$, respectively, to provide both the horizontal (x) and vertical (y) gradient contrast, which ensures that a phase object is equally well‐resolved along all directions in the image.

### 2.2 Metasurface Design and Characterization

Figure 2a shows a schematic of the unit cell of the metasurface, which consists of a periodic array of plasmonic nanorods on top of a silicon nitride waveguiding layer of thickness 150 nm. The metasurface is sandwiched between a 500‐nm thick layer of polymethyl methacrylate (PMMA) as the superstrate and a borosilicate glass substrate. The metallic nanorods, assumed rectangular, have lengths l = 100 nm, widths w = 64 nm, and heights of h = 33 nm. To promote adhesion, a 3‐nm layer of chromium was deposited prior to 30 nm of silver. The unit cell consists of two identical nanorods oriented perpendicularly to each other and positioned diagonally across the unit cell. The unit cell is rectangular with a periodicity of $P_{x}$ = 400 nm along the x‐axis and $P_{y}$ = 370 nm along the y‐axis. The center‐to‐center separation of the nanorods is $P_{x}/4$ along the x‐direction and $P_{y}/4$ along the y‐direction. The design was fabricated using a standard lithographic procedure and the details are outlined in the Methods section. The fabricated metasurfaces were of overall size 300 × 300 μm. Figure 2b shows scanning electron micrographs of a portion of the fabricated metasurface, showing a slight nonuniformity in the dimensions of the nanorods of different unit cells. This can cause random scattering of light which contributes to image noise when the metasurface is used as an image filter.

**FIGURE 2:**
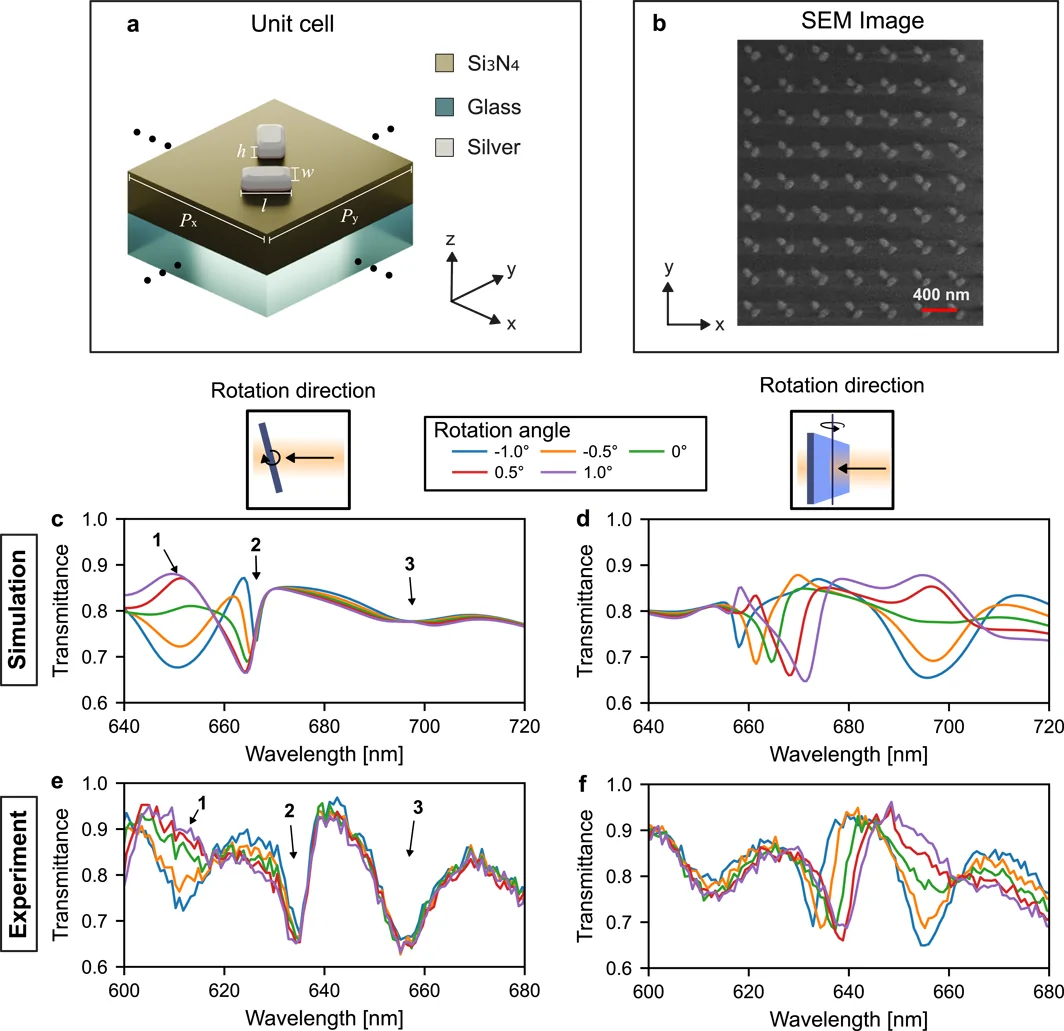
Design and simulation of the metasurface. (a) Schematic of a unit cell of the metasurface. (b) Scanning electron microscope (SEM) images of a portion of the device. (c, d) Simulated and (e, f) experimentally measured transmission spectra at varying incident angles. (c, e) Tilting of the metasurface such that $u_{y}$ is varied while $u_{x}$ is held constant. (d, f) Tilting of the metasurface such that $u_{x}$ is varied while $u_{y}$ is held constant.

The starting point for our design is the chiral guided‐mode optical routing scheme [27, 43, 44], where the arrangement of two orthogonally oriented rectangular scatterers, as seen in Figure 2a, excites guided modes propagating along a single direction determined by the handedness of the incident illumination, resulting in approximately linear spatial dispersion about normal incidence. This contrasts with symmetric resonant waveguide gratings, which couple incident light into standing waves, resulting in parabolic spatial dispersion near normal incidence. As a consequence, the angle‐dependent transmission at wavelengths near resonance exhibits a chirality‐dependent asymmetry about normal incidence. This structure has been demonstrated previously to generate phase gradient contrast, limited to a single axis since only modes propagating along one direction were excited [27]. The key modification in our design is to make the length of the unit cell in the y direction comparable to that in the x direction. As a result, guided modes propagating in the y direction can be excited at a slightly shorter wavelength than those propagating in the x direction. For the guided mode resonance at the shorter wavelength, we observe an asymmetric dispersion along y instead of x, which permits changing the contrast direction by changing the incident wavelength.

Figure 2c–f shows a comparison of simulated and experimentally obtained angle‐dependent transmission spectra with incident circularly polarized light and with no analyzer prior to detection. Numerical results were obtained with full‐wave simulations using the finite element method implemented in COMSOL Multiphysics 6.3 with the Wave Optics module, whereas the experimental results were obtained using a custom benchtop spectroscopy setup. Further details of the simulation and spectroscopy setup are given in the Methods section. Three resonance features labeled 1–3 can be seen in both simulated and experimental results in Figure 2c–f. Note that the simulation results are plotted over a different wavelength range to the experimental results for a clearer comparison, since we found that the resonances observed in the experiments were blue‐shifted compared to the simulations. With a 40 nm offset as shown, the experimental and simulated transmission spectra show good qualitative agreement. This discrepancy is primarily due to the silicon nitride in the experiments having a lower refractive index than that used in the simulations. As shown in Supporting Information S1: Figure S1, decreasing the refractive index to 1.9 from 2.05 in the simulations resulted in spectral positions in agreement with the experiments. This refractive index is within the range observed for silicon nitride deposited using plasma‐enhanced chemical vapor deposition [47].

Figure 2c–e shows the variation of the spectra for rotations corresponding to varying $u_{y}$ values, with $u_{x}$ held fixed at zero, where we see that Feature 1 changes in response, whereas Features 2 and 3 were largely unaffected. Figure 2d–f shows the variation of the spectra for rotations corresponding to varying $u_{x}$ values, with $u_{y}$ held fixed at zero, where now we see that Features 2 and 3 resonances now change in response to different incident angles. This result shows that feature 1 corresponds to the excitation of a guided mode that propagates along y, while Features 2 and 3 correspond to the excitation of guided modes that propagate along x. This agrees with our expectation that the guided mode coupled along y should occur at a shorter wavelength than the guided mode of the same order coupled along x. The apparent difference between the simulated and experimental spectra at Feature 3 in Figure 2c–e was due to a slight rotation of around 1° in the orthogonal tilt direction in the experiments, which from Figure 2d,f, produces the necessary change in the lineshape. Furthermore, by reversing the handedness of the incident circularly polarized light, the spectra at positive angles mapped to those at negative angles, confirming the polarization routing behavior. This result is shown in Supporting Information S1: Figure S2. In the spectra, Feature 2 can be attributed to a transverse‐magnetic guided mode, whereas the other two features are associated with transverse‐electric guided modes, deduced by applying the weakly perturbed slab waveguide model [48].

Figure 3 shows the simulated and experimental magnitudes of the CTFs with incident linear polarization along x and LCP and RCP output analyzers. As mentioned above, the simulations were performed using COMSOL Multiphysics 6.3 and the experimental results were obtained using a custom benchtop back focal plane imaging setup, both described in the Methods section. In contrast to the results obtained in Figure 2, where the metasurface was illuminated with circularly polarized light, we switched to the polarization multiplexing scheme using incident linearly polarized light and filtered output LCP and RCP light obtained using a quarter waveplate and a polarization sensitive camera for the simultaneous acquisition of RCP and LCP images as shown in Figure 1. Although the operating principle of the polarization‐routing metasurface assumed incident circular polarization, we can use the fact that linearly polarized light consists of an even superposition of LCP and RCP light to obtain gradient contrast reversed images simultaneously. For the simulations, we observed vertical contrast at λ= 650 nm, and horizontal contrast at λ= 695 nm. For the experiments, we observed vertical (y) contrast at λ= 613 nm and horizontal (x) contrast at λ= 656 nm. Although the simulation results were obtained at a single wavelength, the experimental results used a quasi‐monochromatic source with a bandwidth of 6 nm full width at half‐maximum.

**FIGURE 3:**
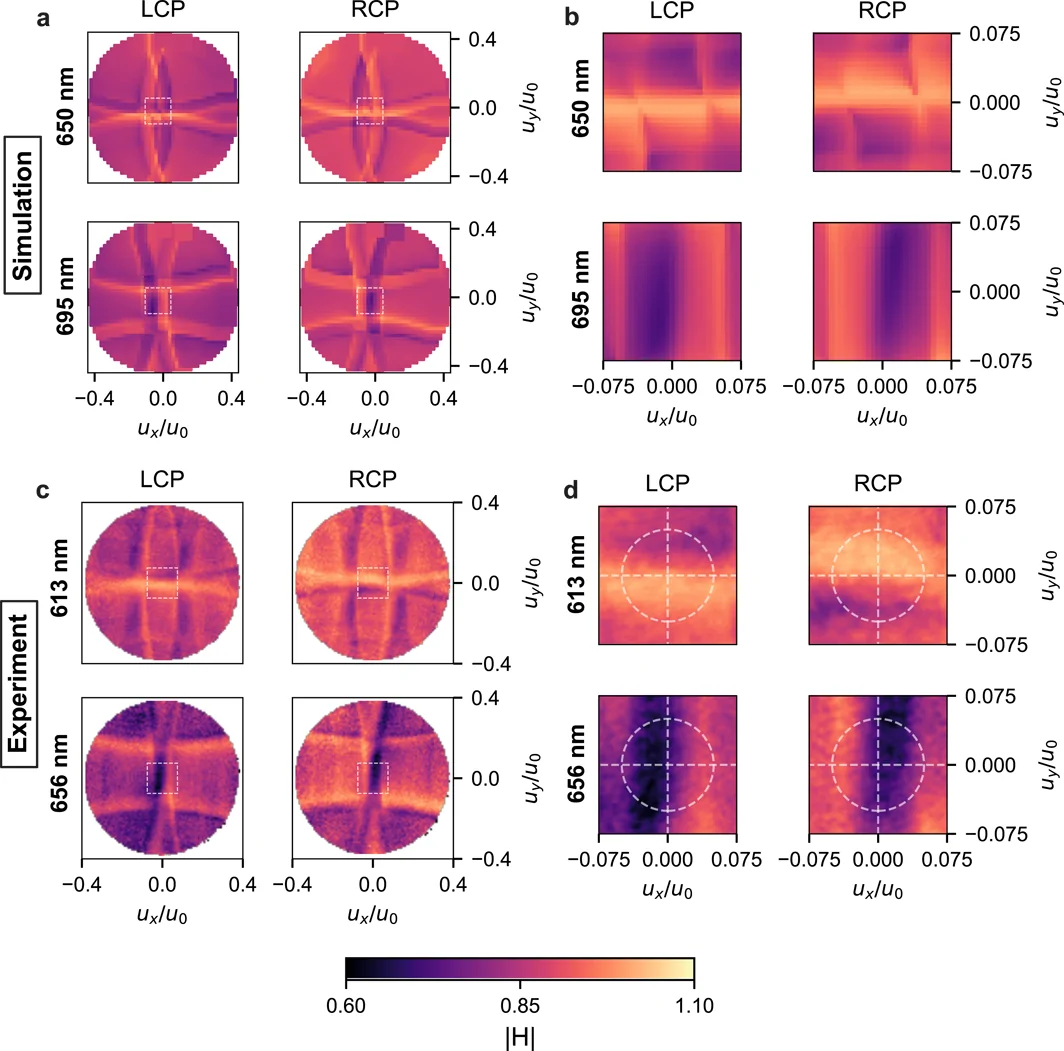
Polarization and wavelength multiplexed coherent transfer functions. (a, b) Simulated and (c, d) experimentally measured absolute values of the CTFs of the metasurface for two operating wavelengths that give vertical (top row) and horizontal (bottom row) gradient contrast. Panels (a, c) are plots over a large NA, whereas panels (b, d) are plots over a small NA, as indicated by the dashed squares in (a) and (c). Here, the horizontal and vertical axes are normalized spatial frequencies, $u_{x}/u_{0}$ and $u_{y}/u_{0}$, where $u_{0}=1/\lambda$.

Figure 3a,c shows the simulated and experimentally obtained CTFs over a numerical aperture (NA) of 0.4, where similar features can be observed in both the simulation and experiment. Note here that the value of |H| can exceed 1 due to polarization conversion. Guided mode resonances can be attributed to the sharp features observed in the plots due to their strong sensitivity to the angle of incidence. At the vertical DPC wavelength, two approximately horizontal curved features meet at the center, whereas at the horizontal DPC wavelength, two vertical curved features meet at the center. We see that one branch has lower transmission than the other, and this is switched upon changing the handedness of the circular polarization analyzer. Auxiliary features perpendicular to the main features are observed at oblique angles due to guided mode resonances coupled in the orthogonal direction.

Figure 3b,d shows magnified plots of the same CTFs near normal incidence, showing the desired responses necessary for two‐axis DPC. Due to the angular selectivity of guided mode resonances, this optical response is restricted to a relatively limited NA. The circled regions in Figure 3d indicate the effective numerical aperture of 0.05. Using this NA, the spatial resolution of our system was estimated to be 6.6 μm at 656 nm using Abbe's definition of resolution: λ/(2NA). The maximum contrast of the transmittance values between positive and negative angles was measured to be 0.25 at 613 nm and 0.4 at 656 nm. The simulated phase of the CTFs is shown in Supporting Information S1: Figure S3, where they are seen to be only slowly varying over the range of interest. Although this was not confirmed experimentally, we can infer that the fabricated device also satisfies this condition from the correspondence between the other simulated and experimental results.

### 2.3 Demonstration of QPM

We first tested the quantitative phase imaging performance of our metasurface‐based system using a reflective liquid crystal spatial light modulator (SLM) (Holoeye Pluto‐VIS‐001) to generate known phase distributions. The setup is described in the Methods section. To calibrate the phase values displayed by the SLM, we used the polarization‐dependent properties of the SLM, detailed in Supporting Information S1: Supplementary Information 4. The quasi‐monochromatic light source and polarization setup were the same as that used for measuring the angle‐dependence. We found that the OPL provided by the SLM is effectively independent of wavelength in the wavelength range considered, as evidenced by the result in Supporting Information S1: Figure S4, which shows that the SLM phase at 656 nm can be determined using the phase at 613 nm by applying Equation (2). In the following, we report the phase at λ= 656 nm.

Figure 4 shows the results of the quantitative phase imaging of sinusoidal star targets with varying phase excursions. The square regions shown correspond to a width of 233 μm at the plane of the metasurface. Figure 4a shows plots of the polarization images obtained from the LCP and RCP channels of the field obtained simultaneously using a polarization‐sensitive camera, shown at the two operating wavelengths for a star target with a 1.5 rad phase excursion. Due to the background intensity noise, the modulations produced by the metasurface are difficult to perceive visually. Using the sum of the LCP and RCP channels, we calculate the background intensity $I_{\text{background}}\left(\rho \right)=I_{+}\left(\rho \right)+I_{-}\left(\rho \right)$, shown in Figure 4b. In the background images, the weak gradient features observed in Figure 4a are no longer prominent in Figure 4b, and any contrast of the object not due to the effects of the metasurface is isolated. Meanwhile, the DPC images shown in Figure 4c isolate the effects of the metasurface, showing clear directional phase gradient contrast, without the common intensity variation between the two channels.

**FIGURE 4:**
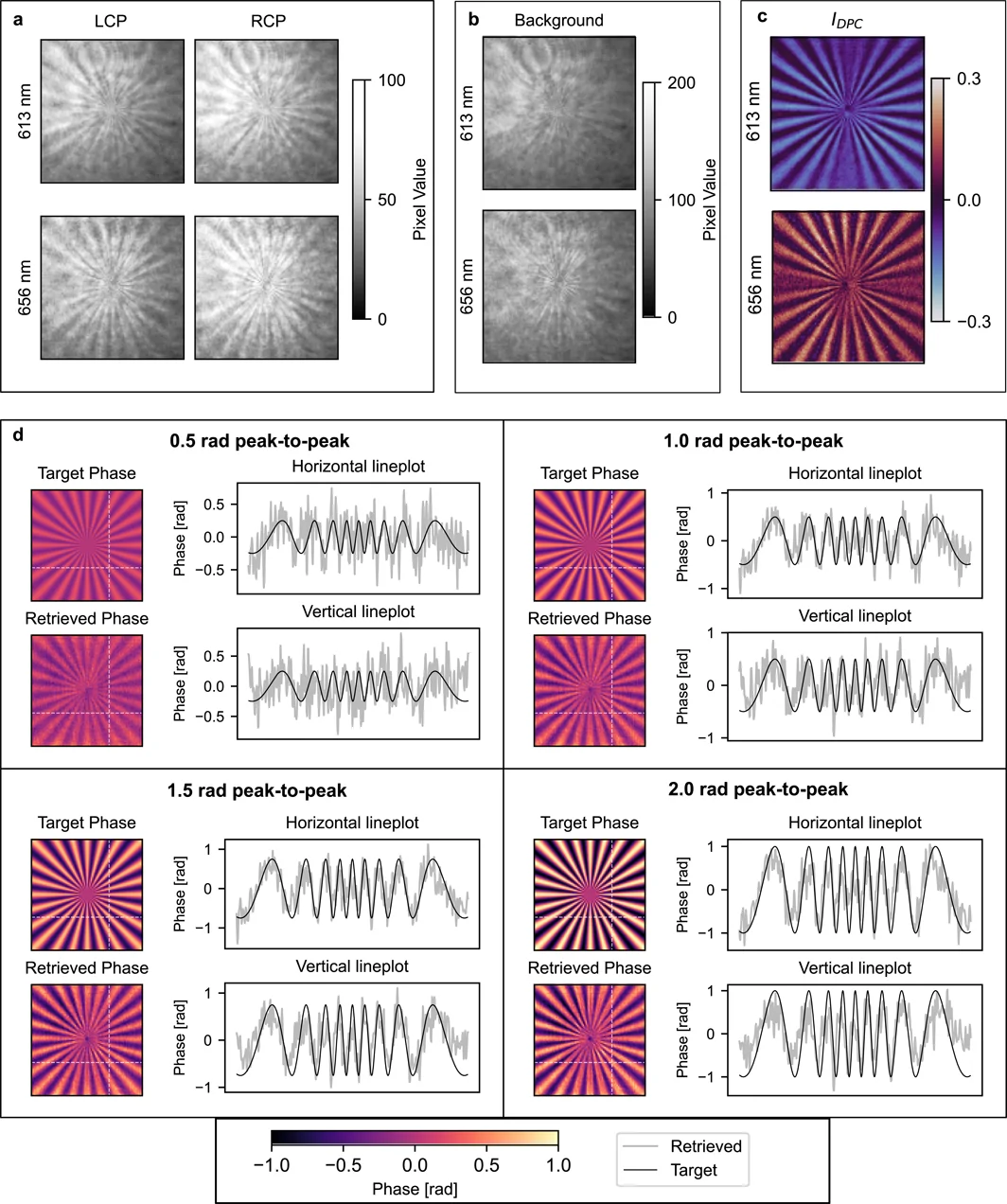
Quantitative phase imaging of star targets. (a) Filtered intensity measurements obtained with a quarter waveplate and a polarization‐sensitive camera. (b) Background images obtained by summing images at the two polarizations. (c) DPC images obtained by subtracting the two polarizations and dividing by the background. (d) Comparison of the retrieved phase with the target phase projected on the SLM for the same target with varying phase excursions, as labeled in each cell. The square regions correspond to a physical size of 233 × 233 µ $m^{2}$.

Figure 4d shows the comparison of the retrieved phase with the target phase projected from the SLM to validate our method. To determine the phase, the value of the regularization parameter was held constant at 0.005 and the experimentally measured CTFs were used to calculate the DPC transfer functions. The order of magnitude of the regularization parameter was determined as the maximum value before the mean‐filtered retrieved phase excursion started to decrease in amplitude. Further decreasing the regularization parameter causes out of band noise to be amplified. Although this could be removed by digital filtering, this would require additional computation. Thus, this optimal value grants the best accuracy of the retrieved phase with the least amount of high frequency, out of band noise, permitting the data to be used without further processing. The retrieved phase shows that each spoke in the image is approximately identical, revealing that our two‐axis phase retrieval is isotropic. The lineplots show that the retrieved phase is consistent with the target phase for a range of spatial frequencies. For increasing phase excursions, the signal‐to‐noise ratio improves due to greater gradient contrast over the spatial noise.

Finally, we applied our metasurface to imaging unstained biological cells, demonstrating its applicability to practical microscopy. We imaged HeLa (cervical cancer) cells grown on a coverslip, shown in Figure 5, with the preparation procedure outlined in the Methods section. A schematic showing the arrangement of components for imaging the cell samples is shown in Figure 5a. The sample was placed in the same setup used to image the SLM phase targets, with the SLM disabled such that it functioned as a flat mirror. The sample was positioned in the front focal plane of a microscope objective and the metasurface was placed immediately after the sample. Shifting the metasurface away from the object plane does not affect the spatial frequency response of the device. Figure 5b shows the retrieved phase, calculated with a regularization parameter of 0.005. The range of phase values is consistent with those given in the literature, which also use visible light with similar wavelengths as here [14, 49]. Figure 5c shows the background and DPC images acquired in the process. Again, we see that although the background intensity contains coherent diffraction artifacts, the DPC images are mostly free of these artifacts. Figure 5d shows the comparison of a DIC image obtained with a commercial DIC microscope (Olympus BX60) in transmission mode with a 20 × 0.4NA objective with a synthesized DIC image calculated using the retrieved phase. The synthesized DIC image was calculated by applying the transfer function of a DIC system with a diagonal shear distance of 5 μm to the field with a uniform intensity and the retrieved phase. We observe that although the DIC images maintain significant gradient contrast even for very small features such as the cell organelles, the DPC and phase images obtained with the metasurface have a lower spatial resolution due to the relatively low effective metasurface NA of 0.05.

**FIGURE 5:**
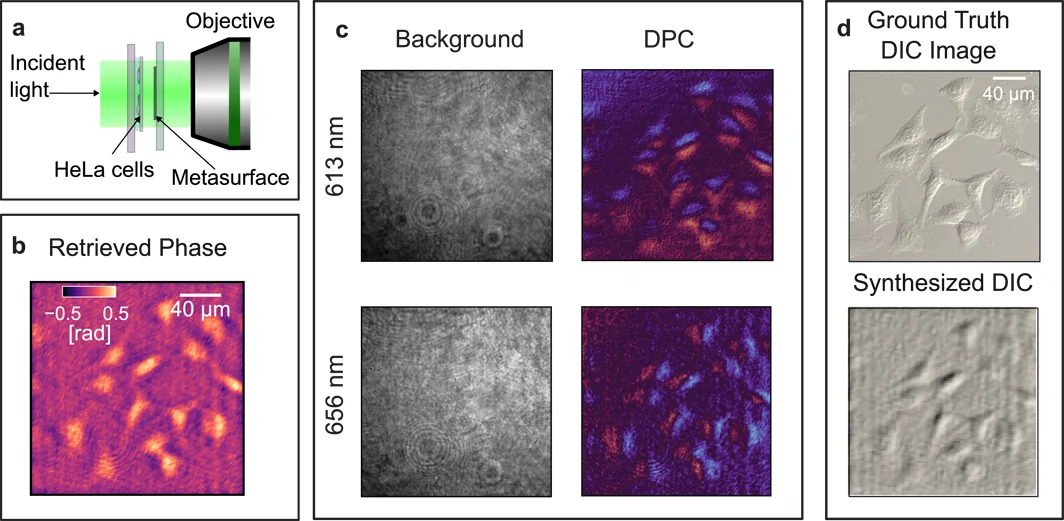
HeLa cell imaging. (a) Configuration for HeLa cell imaging. (b) Retrieved phase image. (c) Background and DPC images. (d) Comparison of an image acquired using a commercial DIC microscope and a synthesized DIC image using the retrieved phase in (a).

## 3 Discussion

We have proposed and experimentally demonstrated quantitative phase imaging using a nonlocal metasurface with wavelength‐ and polarization‐multiplexed spatial filtering functions. Despite the spatially coherent illumination, the resulting DPC and retrieved phase images do not exhibit noise associated with speckle, as the image subtraction process eliminates the background. Although polarization multiplexing enabled a single‐shot capture of two gradient‐reversed images with a polarization‐sensitive camera, wavelength switching was required to capture DPC along orthogonal axes in the image, which was speed restricted due to the software constraints of the tunable filter used with the supercontinuum laser source. Barring this limitation, the next bottleneck for QPM framerate is the speed of the phase retrieval algorithm, which requires only 3 discrete Fourier transform operations, and can be accelerated using faster processors or parallel computing techniques.

The estimated spatial resolution of the phase imaging modality is 6.6 μm based on the 0.05 NA range of spatial frequencies over which gradient contrast can be observed. This limits the features that can be clearly distinguished to features larger than around 10 μm. Consequently, this modality is best suited to imaging features larger than 10 μm, such as the nuclei of mammalian cells (including the HeLa cells studied here), whose morphology is frequently altered in diseased states and routinely used as a diagnostic marker in pathology. We note that this resolution constraint falls only upon the phase imaging modality, and brightfield image obtained by summing the gradient reversed images will have resolution limited by only the imaging optics, allowing smaller features to be viewed, although without the enhanced contrast and quantitative information provided by phase imaging. One method to harness the resolution of the metasurface is to place the device in the detection plane, where the object is magnified and the spatial frequencies are compressed into a smaller range; however, this comes at the cost of phase sensitivity and requires fabricating a larger metasurface to cover the same field of view. This scheme can be explored in future research.

Future work will be aimed at obtaining measurements at a single wavelength or integrating filters to permit single‐shot wavelength multiplexed imaging as well as extending the metasurface NA. We do anticipate, however, that new approaches to engineering the metasurface transfer function will be required.

## 4 Conclusion

In conclusion, we have demonstrated a novel nonlocal metasurface engineered with four distinct spatial frequency responses accessed by tuning the wavelength and polarization for efficient quantitative phase contrast microscopy. To our knowledge, this is the first time such a multiplexing scheme has been demonstrated, and this work opens the path to exploring additional functionalities that can be multiplexed onto a single layer metasurface. We envisage that such metasurfaces can be integrated with other meta‐optical components and advanced light sources to produce imaging devices with unprecedented performance and compact form factors.

## 5 Methods

### 5.1 Simulation

Finite element method frequency domain simulations of the metasurface unit cell were conducted using COMSOL Multiphysics 6.3. A single rectangular unit cell was modeled with Floquet periodic boundary conditions to emulate an infinite lattice and to model plane waves at off‐normal incidences. Input and output port boundaries were placed sufficiently far from the metasurface to accommodate the near fields. The substrate and superstrate were modeled with fixed refractive indexes of 1.5; the refractive index of silicon nitride was modeled using a Sellmeier dispersion model [50], and silver and chromium were modeled with data for bulk materials [51]. At the output port, the complex amplitudes of transmitted LCP and RCP plane waves were computed. To calculate the spectra in Figure 2, the LCP and RCP transmittances were summed, whereas to calculate the CTFs in Figure 3, we calculated the S‐parameters for LCP and RCP transmitted waves.

### 5.2 Fabrication

Silicon nitride was deposited onto borosilicate glass wafer pieces using plasma‐enhanced chemical vapor deposition (Oxford Instruments Plasmalab 100 PECVD) at a temperature of 300°C. A 200‐nm thick layer of polymethyl methacrylate resist was spin‐coated onto the substrate and baked for 3 min at 180°C followed by spin‐coating a water‐soluble conducting polymer (Dischem Discharge H2Ox2). The patterns for the metasurface were defined using electron beam lithography. The resist was developed using a 3:1 solution of IPA:MIBK. 3 nm thick chromium followed by deposition of a 30‐nm thick silver was deposited using electron beam evaporation (Intlvac Nanochrome II), and the sample is placed in an acetone bath overnight for lift‐off. A 500‐nm thick PMMA cover layer was then spin‐coated and baked to form a protective superstrate.

### 5.3 Optical Characterization

The custom setups used for characterizing the angle‐dependent and spectral transmission response (Figure 3) will be described briefly here. Diagrams of the systems and detailed descriptions of each component can be found in Supporting Information S1: Supplementary Information 5.

To measure the transmission spectra at varying incident angles (Figure 2b,c), the sample was illuminated with collimated light from a multimode fiber coupled halogen lamp (OceanOptics HL‐2000‐HP). Before illuminating the sample, a linear polarizer (Thorlabs LPVIS050‐MP2) and a quarter waveplate (Thorlabs AHWP10M‐580) were used to circularly polarize the light. The sample was mounted on a motorized rotation stage (Thorlabs PRM1Z8) to control the tilt of the sample. The transmitted field of view was restricted using an aperture, and the light was coupled into a visible light spectrometer (OceanOptics HR2000+ES) via a multimode optical fiber cable. A bright reference was taken using an unpatterned region of the sample.

To measure the absolute values of the CTFs (Figure 3d), we built a back focal plane imaging setup. The metasurface was illuminated with a focal spot generated by a high numerical aperture objective with its back aperture filled by a collimated, single‐mode beam from a filtered supercontinuum source (NKT SuperK Compact with a SuperK Select acousto‐optic filter). The beam was set to horizontal polarization using a linear polarizer (Thorlabs LPVIS050‐MP2). An imaging objective collected the transmitted light, which then passed through a quarter waveplate (Thorlabs AHWP10M‐580) with its fast axis at 45° to the metasurface axes, and the back focal plane was relayed to a polarization‐sensitive camera (Thorlabs Kiralux Polarization Camera CS505MUP1).

### 5.4 Imaging

The imaging setup used with the metasurface is described in detail and pictured in Supporting Information S1: Supplementary Information 5. A brief description is given here. Collimated light from the filtered supercontinuum source given above was horizontally polarized and expanded using an objective and a lens to illuminate the SLM (Holoeye Pluto‐2). The beam, which may have phase imparted by the SLM, was then demagnified and collimated using a lens and then an objective to illuminate the metasurface. During cell imaging, the SLM was disabled, and the cell sample was placed in the path of this narrow, collimated beam, which ensures sufficient light intensity across the region of interest. After the metasurface, the image of the SLM or sample was relayed to a polarization camera (given above) with an objective and a lens, passing through a quarter waveplate (Thorlabs AHWP10M‐580) with its fast axis at 45° to the metasurface axes before the sensor.

### 5.5 Cell Sample Preparation

22 × 22 mm coverslips were placed in a 35‐mm imaging dish. Coverslips were coated with 0.1 mg/mL Poly‐D‐Lysine (PDL) for 30 min at room temperature. HeLa cells were grown in DMEM (Lonza) supplemented with 10% bovine growth serum (Gibco), 1 × Pen‐Strep (Lonza) at 37°C in 5% CO_2_. Cells were fixed with 4% paraformaldehyde for 15 min at room temperature. After fixation, coverslips were transferred onto glass slides for imaging.

## Author Contributions

**Haiwei Wang:** conceptualization, investigation, writing – original draft, methodology, validation, visualization, writing – review and editing, software, formal analysis, data curation, project administration. **Shikun Ma:** methodology, validation, writing – review and editing, investigation. **Shaban Sulejman:** formal analysis, writing – review and editing, methodology, investigation. **Niken Priscilla:** investigation, writing – review and editing, methodology, formal analysis. **Wendy S. L. Lee:** investigation, methodology, writing – review and editing. **Peter Francis Mathew Elango:** methodology, writing – review and editing, investigation. **Lukas Wesemann:** conceptualization, investigation, supervision, writing – original draft, writing – review and editing, validation, methodology, formal analysis, project administration. **Elizabeth Hinde:** resources, supervision, investigation, writing – review and editing, funding acquisition, project administration. **Ann Roberts:** funding acquisition, investigation, conceptualization, writing – original draft, writing – review and editing, supervision, resources, formal analysis, project administration, validation, methodology.

## Conflicts of Interest

The authors declare no conflicts of interest.

## Supporting information


Supporting Information S1

## Data Availability

The data that support the findings of this study are available from the corresponding author upon reasonable request.
