# Supplementary material for: Wavelength and Polarization Multiplexed Nonlocal Metasurface for Quantitative Phase Microscopy
Source: Nanophotonics. 2026 Jul 1;15(13):e70190. doi: 10.1002/nap2.70190 (PMC13642177; doi:10.1002/nap2.70190)
Supplement: Supplementary file 1 [file NAP2-15-e70190-s001.pdf]

Supplementary information: wavelength and polarization multiplexed nonlocal metasurface for quantitative phase microscopy

Haiwei Wang[1], Shikun Ma[2,3], Shaban B. Sulejman[1], Niken Priscilla[1], Wendy S.L. Lee[1], Peter Francis Mathew Elango[4,5], Lukas Wesemann[1], Elizabeth Hinde[2] and Ann Roberts[1]

[1] ARC Centre of Excellence for Transformative Meta-Optical Systems, School of Physics, The University of Melbourne, Victoria 3010, Australia

[2] School of Physics, The University of Melbourne, Victoria 3010, Australia

[3] Department of Biochemistry and Pharmacology, The University of Melbourne, Victoria 3010, Australia

[4] Functional Materials and Microsystems Research Group and the Mico Nano Research Facility, RMIT University, Melbourne, 3001, Australia

[5] ARC Centre of Excellence for Transformative Meta-Optical Systems, RMIT University, Melbourne, 3001, Australia

Supplementary Information 1: Fourier transforms and angular spectrum

1.1 Two-dimensional Fourier transform

Consider a 2D position space scalar function $f(\boldsymbol{\rho})$ of the position coordinates $\boldsymbol{\rho} = (x, y)$. Its spatial frequency space representation denoted by the function $\tilde{f}(\boldsymbol{u})$ of the spatial frequency coordinates $\boldsymbol{u} = (u_x, u_y)$, is given by the Fourier transform

$$\tilde{f}(\boldsymbol{u}) = \mathcal{F}\{f(\boldsymbol{\rho})\}(\boldsymbol{u}) \equiv \int f(\boldsymbol{\rho}) e^{-i2\pi \boldsymbol{u}\cdot\boldsymbol{\rho}} d^2\boldsymbol{\rho} = \int f(x, y) e^{-i2\pi(u_x x + u_y y)} dx dy, \quad \text{(SE1)}$$

where i is the imaginary unit, and $\mathcal{F}$ is the Fourier transform operator. The inverse Fourier transform recovers the original function from its Fourier transform, it is defined as

$$f(\boldsymbol{\rho}) = \mathcal{F}^{-1}\{\tilde{f}(\boldsymbol{u})\}(\boldsymbol{\rho}) \equiv \int \tilde{f}(\boldsymbol{u}) e^{i2\pi \boldsymbol{u}\cdot\boldsymbol{\rho}} d^2\boldsymbol{u} = \int \tilde{f}(u_x, u_y) e^{i2\pi(u_x x + u_y y)} du_x du_y. \quad \text{(SE2)}$$

1.2 Angular spectrum of scalar fields

The Fourier transform formalism is used to describe the propagation of a scalar field in a homogeneous medium. A scalar field $\psi(\boldsymbol{\rho}, z)$ is written as a function of transverse coordinates $\boldsymbol{\rho} = (x, y)$, and a longitudinal coordinate z. We assume the scalar field is a solution of the Helmholtz equation

$$(\nabla^2 + 4\pi^2 u_0^2)\psi(\boldsymbol{\rho}, z) = 0, \tag{SE3}$$

Where $\nabla^2 = \frac{\partial^2}{\partial x^2} + \frac{\partial^2}{\partial y^2} + \frac{\partial^2}{\partial z^2}$ is the Laplace operator, and u_0 is the spatial frequency of the wave, given by the frequency of the oscillation divided by the wave propagation speed. Assuming that the field propagates in the $+z$ direction only, $\psi(\boldsymbol{\rho}, z)$ can be determined if $\psi(\boldsymbol{\rho}, 0)$ is known, with

$$\psi(\boldsymbol{\rho}, z) = \int \tilde{\psi}(\boldsymbol{u}, 0) e^{i2\pi \boldsymbol{u}\cdot\boldsymbol{\rho}} e^{i2\pi u_z(\boldsymbol{u})z} d^2\boldsymbol{u}, \tag{SE4}$$

where

$$\tilde{\psi}(\boldsymbol{u}, 0) = \int \psi(\boldsymbol{\rho}, 0) e^{-i2\pi \boldsymbol{u}\cdot\boldsymbol{\rho}} d^2\boldsymbol{\rho}, \tag{SE5}$$

is the angular spectrum of the field, and

$$u_z(\boldsymbol{u}) = \sqrt{u_0^2 - |\boldsymbol{u}|^2} = \sqrt{u_0^2 - u_x^2 - u_y^2}. \tag{SE6}$$

Thus, the propagation of the field is described by a superposition of plane waves at different incident angles. $\tilde{\psi}(\boldsymbol{u}, 0)$ is called the angular spectrum since it corresponds to the amplitude of a plane wave propagating in the direction given by $\boldsymbol{s} = \left(u_x/u_0,\ u_y/u_0,\ u_z/u_0\right)$ when $|\boldsymbol{u}| \leq u_0$. For the case $|\boldsymbol{u}| > u_0$, the field no longer propagates along the z-direction but decays exponentially.

Diffraction of a scalar field by a thin transmission mask can be described in real space. Given an incident field $\psi_{\mathrm{i}}(\boldsymbol{\rho})$ and a thin mask $t(\boldsymbol{\rho})$, the transmitted field immediately after the mask $\psi_{\mathrm{t}}(\boldsymbol{\rho})$ is given by $\psi_{\mathrm{t}}(\boldsymbol{\rho}) = t(\boldsymbol{\rho})\psi_{\mathrm{i}}(\boldsymbol{\rho})$. The field after the transmission mask can be determined by propagating $\psi_{\mathrm{t}}(\boldsymbol{\rho})$.

1.3 Angular spectrum of electromagnetic fields

An electromagnetic field is specified by two vector fields: the electric field $\boldsymbol{E}(\boldsymbol{\rho}, z) = (E_x, E_y, E_z)$, and the magnetic field $\boldsymbol{H}(\boldsymbol{\rho}, z) = (H_x, H_y, H_z)$. In a homogeneous medium, each Cartesian component of the electric and magnetic field satisfies the Helmholtz equation (SE3), and each component can be propagated using SE4.

Diffraction of an electromagnetic field by a thin mask differs from that of the scalar field. This stems from the requirement that each plane wave component must be transverse to its direction of propagation. Given an incident vector field $\boldsymbol{E}_\mathrm{i}(\boldsymbol{\rho})$, and scalar transmission mask $t(\boldsymbol{\rho})$, the transmitted field is given by $\boldsymbol{E}_\mathrm{t}(\boldsymbol{\rho}) = t(\boldsymbol{\rho})\boldsymbol{E}_\mathrm{i}(\boldsymbol{\rho})$. To satisfy the transverse wave condition, the angular spectrum of the transmitted field will not be the Fourier transform of each Cartesian component, it is instead[1]

$$\widetilde{\boldsymbol{E}}_\mathrm{t}(\boldsymbol{u}) = \left(\mathbb{I} - \frac{\boldsymbol{u}\boldsymbol{u}}{u_0^2}\right)\mathcal{F}\{t(\boldsymbol{\rho})\boldsymbol{E}_\mathrm{i}(\boldsymbol{\rho})\}(\boldsymbol{u}), \tag{SE7}$$

Where $\mathbb{I}$ is the identity tensor. In terms of Cartesian components, this is

$$\begin{pmatrix} \tilde{E}_{\mathrm{t},x}(\boldsymbol{u}) \\ \tilde{E}_{\mathrm{t},y}(\boldsymbol{u}) \\ \tilde{E}_{\mathrm{t},z}(\boldsymbol{u}) \end{pmatrix} = \begin{pmatrix} 1 - \frac{u_x^2}{u_0^2} & -\frac{u_x u_y}{u_0^2} & -\frac{u_x u_z}{u_0^2} \\ -\frac{u_x u_y}{u_0^2} & 1 - \frac{u_y^2}{u_0^2} & -\frac{u_y u_z}{u_0^2} \\ -\frac{u_x u_z}{u_0^2} & -\frac{u_y u_z}{u_0^2} & 1 - \frac{u_z^2}{u_0^2} \end{pmatrix} \begin{pmatrix} \mathcal{F}\{t(\boldsymbol{\rho})E_{\mathrm{i},x}(\boldsymbol{\rho})\}(\boldsymbol{u}) \\ \mathcal{F}\{t(\boldsymbol{\rho})E_{\mathrm{i},y}(\boldsymbol{\rho})\}(\boldsymbol{u}) \\ \mathcal{F}\{t(\boldsymbol{\rho})E_{\mathrm{i},z}(\boldsymbol{\rho})\}(\boldsymbol{u}) \end{pmatrix}. \tag{SE8}$$

Thus, given an incident plane wave with amplitude E_0 and uniform polarization $\hat{\boldsymbol{e}}_\mathrm{in}$ as in the main text, the diffracted angular spectrum is

$$\widetilde{\boldsymbol{E}}_\mathrm{t}(\boldsymbol{u}) = E_0 \tilde{t}(\boldsymbol{u}) \left(\mathbb{I} - \frac{\boldsymbol{u}\boldsymbol{u}}{u_0^2}\right)\hat{\boldsymbol{e}}_\mathrm{in}, \tag{SE9}$$

which shows that the diffracted plane wave components will not have the same polarization as the incident polarization. However, for small diffraction angles, which is assumed in the main text, $\boldsymbol{u}\boldsymbol{u}/u_0^2$ is approximately zero, then, the diffracted plane waves composing the object field will have the same polarization as the incident polarization.

Supplementary Information 2: Derivation of the intensity transfer functions

2.1 Calculation of the phase transfer function

The intensity of a coherent field generated by a plane wave at normal incidence with intensity $I_0 = |E_0|^2$ illuminating an object $t(\boldsymbol{\rho})$ and after passing through an optical system described by the coherent transfer function $H(\boldsymbol{u})$ is given by

$$I(\boldsymbol{\rho}) = I_0 \left| \iint d^2\boldsymbol{u}\, H(\boldsymbol{u}) \exp(i2\pi\boldsymbol{\rho}\cdot\boldsymbol{u}) \iint d^2\boldsymbol{\rho}'\, t(\boldsymbol{\rho}') \exp(-i2\pi\boldsymbol{\rho}'\cdot\boldsymbol{u}) \right|^2. \tag{SE10}$$

Expanding the absolute value squared:

$$\begin{aligned} I(\boldsymbol{\rho}) = I_0 &\iint d^2\boldsymbol{u}\, H(\boldsymbol{u}) \exp(i2\pi\boldsymbol{\rho}\cdot\boldsymbol{u}) \iint d^2\boldsymbol{\rho}'\, t(\boldsymbol{\rho}') \exp(-i2\pi\boldsymbol{\rho}'\cdot\boldsymbol{u}) \\ \times &\iint d^2\boldsymbol{u}'\, H^*(\boldsymbol{u}') \exp(-i2\pi\boldsymbol{\rho}\cdot\boldsymbol{u}') \iint d^2\boldsymbol{\rho}''\, t^*(\boldsymbol{\rho}'') \exp(i2\pi\boldsymbol{\rho}''\cdot\boldsymbol{u}'). \end{aligned} \tag{SE11}$$

We apply the weak phase approximation, where

$$t(\boldsymbol{\rho}')t^*(\boldsymbol{\rho}'') \approx 1 + i[\phi(\boldsymbol{\rho}') - \phi(\boldsymbol{\rho}'')]. \tag{SE12}$$

We compute these three terms separately, for the first term

$$\begin{aligned} I_{\mathrm{BG}}(\boldsymbol{\rho}) = I_0 \iint \dots \iint d^2\boldsymbol{u}\, d^2\boldsymbol{u}'\, d^2\boldsymbol{\rho}' d^2\boldsymbol{\rho}'' H(\boldsymbol{u}) H^*(\boldsymbol{u}') \exp[i2\pi\boldsymbol{\rho}\cdot(\boldsymbol{u}-\boldsymbol{u}')] \\ \times \exp(-i2\pi\boldsymbol{\rho}'\cdot\boldsymbol{u}) \exp(i2\pi\boldsymbol{\rho}''\cdot\boldsymbol{u}'), \end{aligned} \tag{SE13}$$

$$I_{\mathrm{BG}}(\boldsymbol{\rho}) = I_0 |H(0)|^2. \tag{SE14}$$

For the second term

$$\begin{aligned} I_{\mathrm{Ph}}(\boldsymbol{\rho}) = iI_0 \iint \dots \iint d^2\boldsymbol{u}\, d^2\boldsymbol{u}_{\mathrm{d}}\, d^2\boldsymbol{\rho}' d^2\boldsymbol{\rho}'' H(\boldsymbol{u}) H^*(\boldsymbol{u}-\boldsymbol{u}_{\mathrm{d}}) \exp(i2\pi\boldsymbol{\rho}\cdot\boldsymbol{u}_{\mathrm{d}}) \\ \times \exp(-i2\pi\boldsymbol{\rho}'\cdot\boldsymbol{u}) \exp[i2\pi\boldsymbol{\rho}''\cdot(\boldsymbol{u}-\boldsymbol{u}_{\mathrm{d}})]\, [\phi(\boldsymbol{\rho}') - \phi(\boldsymbol{\rho}'')], \end{aligned} \tag{SE15}$$

where we transformed the integration variable $\boldsymbol{u}'$ to $\boldsymbol{u}_{\mathrm{d}} = \boldsymbol{u} - \boldsymbol{u}'$. Integrating over $\boldsymbol{u}$ and using the fact that $\phi(\boldsymbol{\rho})$ is a real function so $\tilde{\phi}^*(\boldsymbol{u}) = \tilde{\phi}(-\boldsymbol{u})$

$$I_{\mathrm{Ph}}(\boldsymbol{\rho}) = iI_0 \iint d^2\boldsymbol{u}_{\mathrm{d}} \exp(i2\pi\boldsymbol{\rho}\cdot\boldsymbol{u}_{\mathrm{d}})\,[H(\boldsymbol{u}_{\mathrm{d}})H^*(0) - H(0)H^*(-\boldsymbol{u}_{\mathrm{d}})]\tilde{\phi}(\boldsymbol{u}_{\mathrm{d}}). \tag{SE16}$$

Therefore, we see that the Fourier spectrum of the intensity can be written as

$$\tilde{I}(\boldsymbol{u}) = I_0|H(0)|^2\delta^2(\boldsymbol{u}) + H_\phi(\boldsymbol{u})\tilde{\phi}(\boldsymbol{u}), \tag{SE17}$$

where

$$H_\phi(\boldsymbol{u}) = iI_0[H^*(0)H(\boldsymbol{u}_{\mathrm{d}}) - H(0)H^*(-\boldsymbol{u}_{\mathrm{d}})] \tag{SE18}$$

is the phase transfer function.

2.2 Calculation of the DPC transfer function

The DPC image is calculated using two images $I_+(\boldsymbol{\rho})$ and $I_-(\boldsymbol{\rho})$ filtered with CTFs $H_+(\boldsymbol{u})$ and $H_-(\boldsymbol{u})$, respectively. The sum of the Fourier spectra of the intensities is

$$\tilde{I}_+(\boldsymbol{u}) + \tilde{I}_-(\boldsymbol{u}) = I_0(|H_+(0)|^2 + |H_-(0)|^2)\delta^2(\boldsymbol{u}) + \left[H_{\phi,+}(\boldsymbol{u}) + H_{\phi,-}(\boldsymbol{u})\right]\tilde{\phi}(\boldsymbol{u}). \tag{SE19}$$

When $H_{\phi,+}(\boldsymbol{u}) = -H_{\phi,-}(\boldsymbol{u})$ is satisfied, the sum of the images will not contain any phase contrast information. Note that this condition does not require the CTFs $H_+(\boldsymbol{u})$ and $H_-(\boldsymbol{u})$ to be perfect mirror images of each other. The difference of the Fourier spectra of the intensities is

$$\tilde{I}_+(\boldsymbol{u}) - \tilde{I}_-(\boldsymbol{u}) = I_0(|H_+(0)|^2 - |H_-(0)|^2)\delta^2(\boldsymbol{u}) + \left[H_{\phi,+}(\boldsymbol{u}) - H_{\phi,-}(\boldsymbol{u})\right]\tilde{\phi}(\boldsymbol{u}). \tag{SE20}$$

The Fourier spectrum of the DPC intensity calculated using Eq. 4 in the main text is

$$\tilde{I}_{\mathrm{DPC}}(\boldsymbol{u}) = \frac{|H_+(0)|^2 - |H_-(0)|^2}{|H_+(0)|^2 + |H_-(0)|^2}\delta^2(\boldsymbol{u}) + \frac{H_{\phi,+}(\boldsymbol{u}) - H_{\phi,-}(\boldsymbol{u})}{|H_+(0)|^2 + |H_-(0)|^2}\tilde{\phi}(\boldsymbol{u}). \tag{SE21}$$

The first term is a DC offset, which is physically irrelevant in phase retrieval and can be neglected, while the second term contains the phase information. The DPC transfer function in Eq. 5 of the main text is hence given by

$$H_{\mathrm{DPC}}(\boldsymbol{u}) = \frac{H_{\phi,+}(\boldsymbol{u}) - H_{\phi,-}(\boldsymbol{u})}{|H_+(0)|^2 + |H_-(0)|^2}. \tag{SE22}$$

Supplementary Information 3: Additional metasurface characterization data

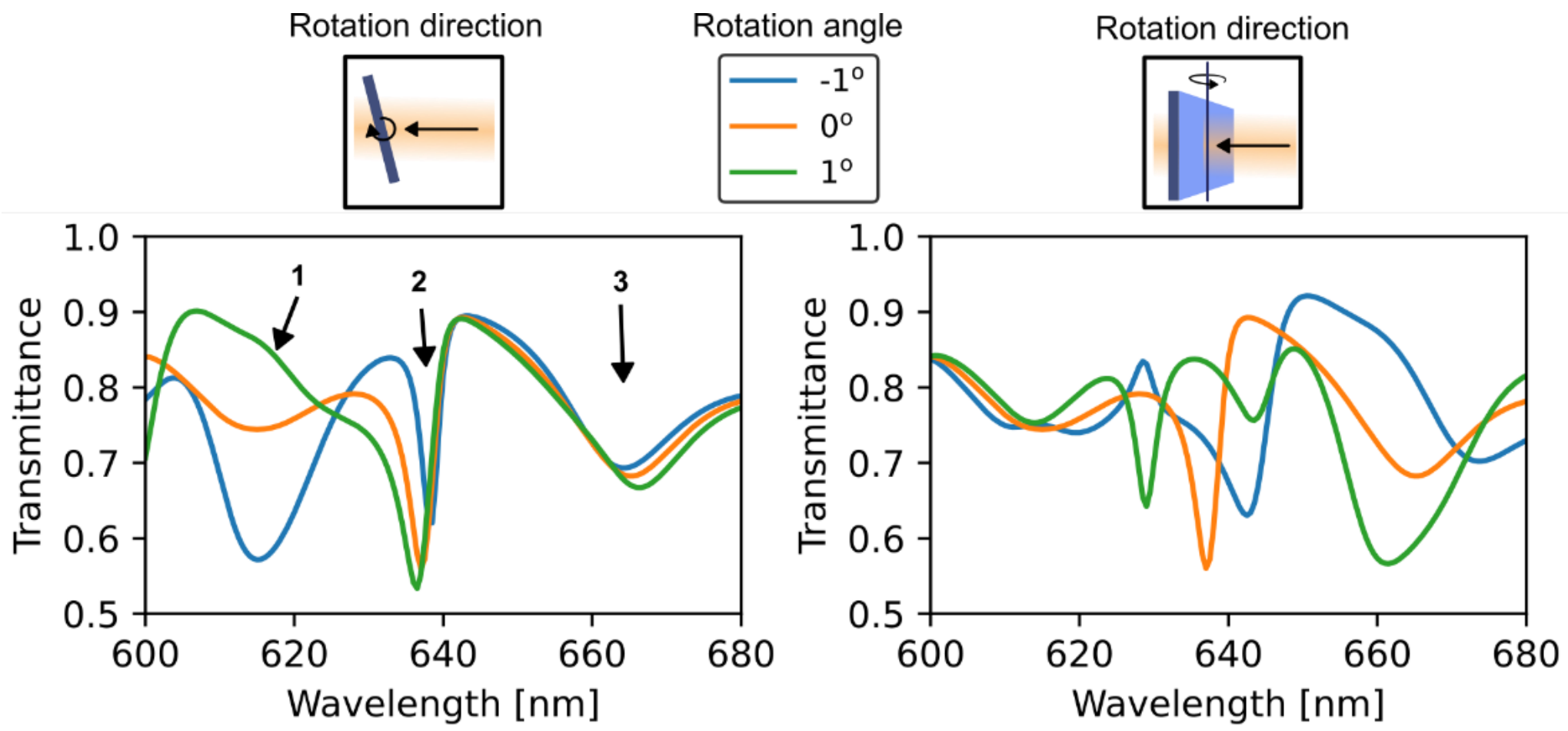

Supplementary Figure 1: Simulated angle-dependent transmission spectra with the refractive index of silicon nitride decreased to 1.9, showing blueshifts of the simulated resonances seen in Figure 2 of the main text to agree with the measured spectra.

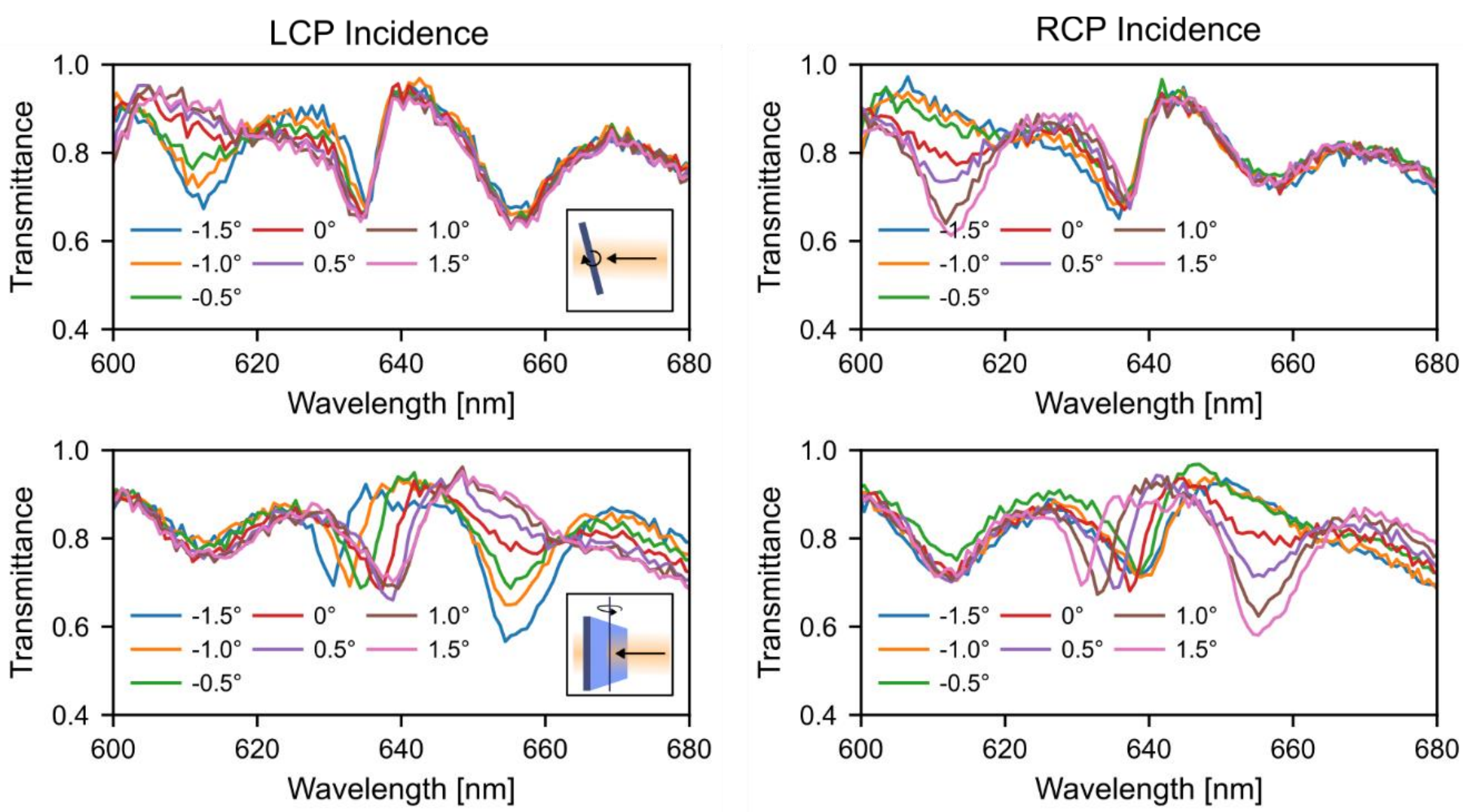

Supplementary Figure 2: Transmission spectra measurements at varying incident angles for LCP vs. RCP incident light.

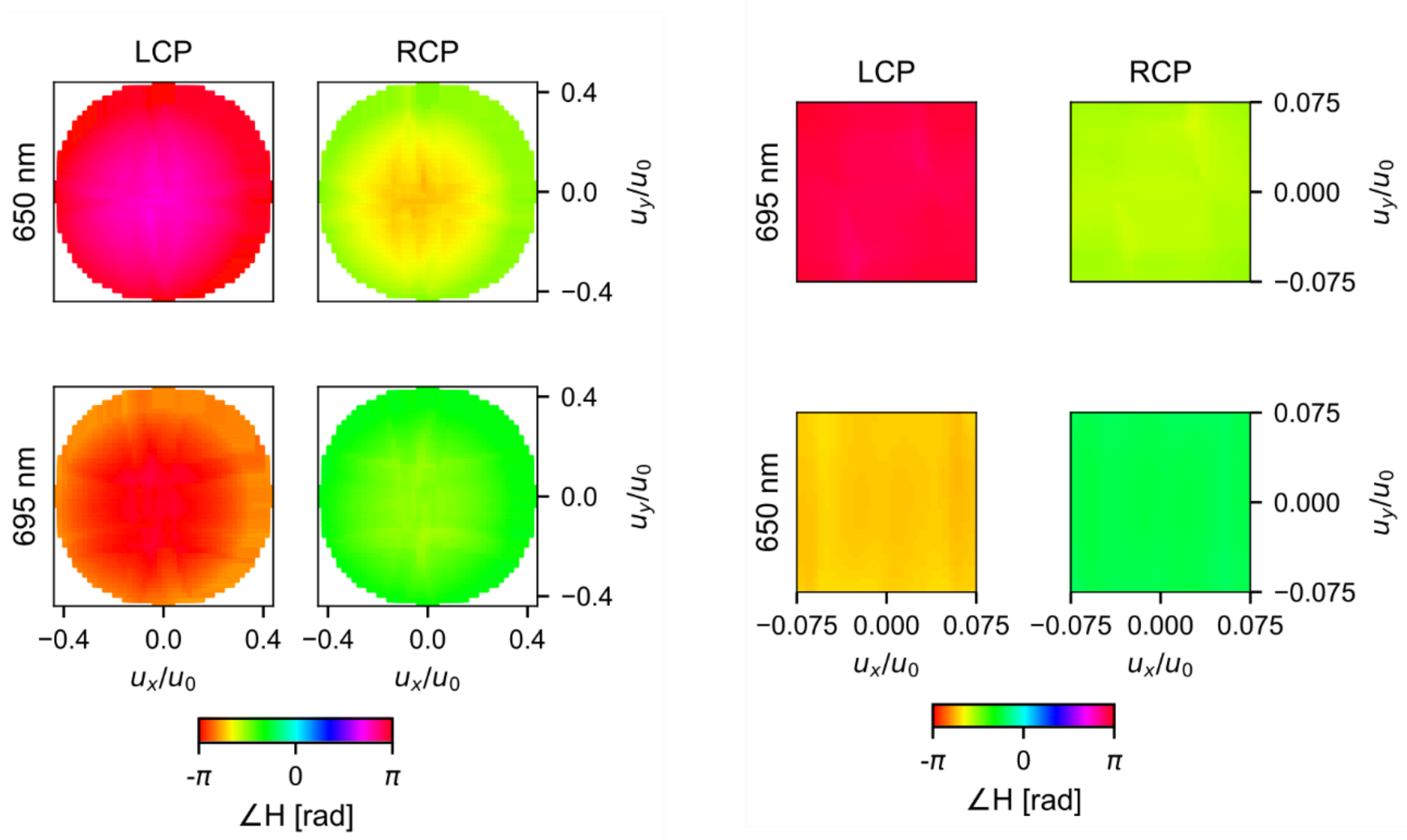

Supplementary Figure 3: Numerically calculated phase of the coherent transfer function.

Supplementary Information 4: Calibrating the phase response of the SLM

The Jones matrix of SLM is expressed as

$$M(\phi) = \begin{pmatrix} e^{i\phi} & 0 \\ 0 & 1 \end{pmatrix}, \tag{SE23}$$

where the first component is horizontal polarization, the horizontal plane corresponds to the plane of rotation of the liquid crystal molecules, hence a variable, voltage-dependent phase shift is applied to this component

$$\phi(V) = \frac{2\pi[n_e(V) - n_o](2d)}{\lambda_0}, \tag{SE24}$$

where $n_e(V)$ is the variable extraordinary refractive index, n_o is the ordinary refractive index, d is the thickness of the liquid crystal layer, and λ_0 is the vacuum wavelength. A factor of two multiplies the thickness because light must make a round trip through the liquid crystal layer to be reflected in a reflection-mode SLM.

With input diagonal polarization given by the Jones vector

$$\frac{1}{\sqrt{2}}\binom{1}{1}.$$

The output polarization is

$$\frac{1}{\sqrt{2}}\binom{e^{i\phi}}{1}.$$

The dot product with the perpendicular diagonal polarization is

$$\frac{1}{2}\left(e^{i\phi}-1\right).$$

The resulting intensity is the modulus-squared of this

$$\frac{I(\phi)}{I_0}=\frac{1}{4}\left(e^{i\phi}-1\right)\left(e^{-i\phi}-1\right)=\frac{1}{2}(1-\cos\phi)=\sin^2\frac{\phi}{2}. \tag{SE25}$$

I_0 is measured as the maximum intensity value for all pixel values displayed. Using the intensity modulation measurements, we calibrate the phase response. Phase unwrapping was applied to obtain phase values above π.

Supplementary Figure 4 shows the measured phase for different pixel values displayed on the SLM obtained with two different wavelengths. The phase response is most linear at the middle of the range. We see that the slope of the curve for 610 nm is slightly steeper than 656 nm, indicating that light at 610 nm experience stronger phase shifts. By applying the wavelength correction factor according to Eq. 2 of the main text and adding a constant offset, the corrected phase values at 610 nm match well with that at 656 nm.

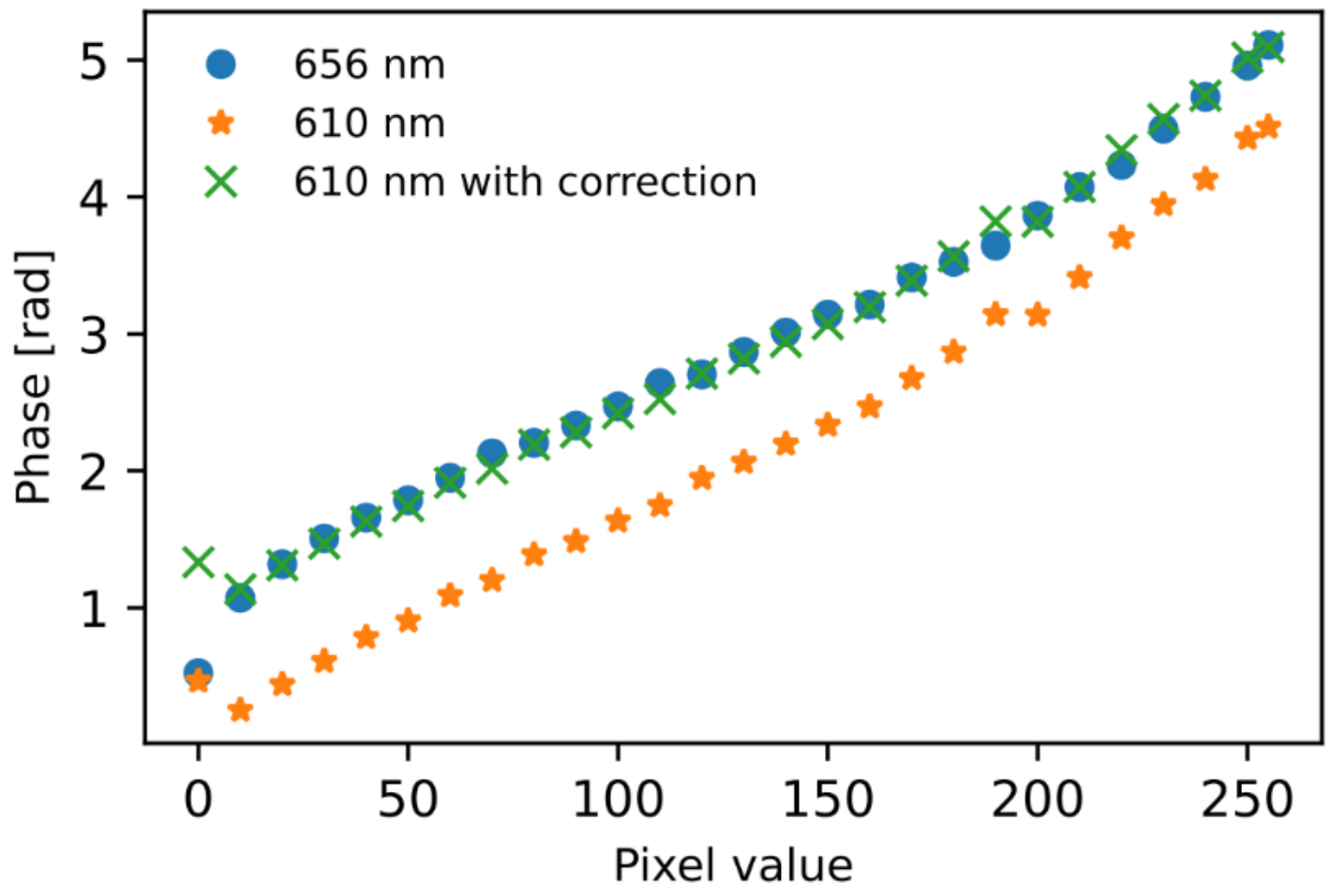

Supplementary Figure 4: SLM phase calibration curve.

Supplementary Information 5: Description of optical setups

The spectroscopy setup is depicted in Supplementary Figure 5. A fiber-coupled output from an OceanOptics HL-2000-HP halogen lamp was collimated using an Olympus UPlanF 4x 0.13NA objective (OL1), producing a beam with a diameter around 1cm. Before illuminating the metasurface, a Thorlabs LPVISE100-A linear polarizer (LP) and a Thorlabs AQWP10M-580 achromatic quarter waveplate (QWP) were placed with the fast axis of the QWP oriented at 45° to the polarization axis of the LP to create circular polarization. The circularly polarized light then illuminated the sample which was mounted on a rotation stage to measure the spectra at varying incident angles. After the sample, a 50x Nikon CFI60 TU Plan 0.55NA objective (OL2) with a long working distance of 11 mm was used to magnify the metasurface area - the relatively long working distance was required to permit rotation of the sample. A Thorlabs LA1131-A lens (L1) with a focal length of 50 mm was placed to collimate the light collected by the objective. The image of the sample was conjugated onto an aperture to limit the subsequently collected light to only that transmitted through the metasurface. The light was then split equally into two paths by a Thorlabs CM1BS013 non-polarizing beamsplitter cube (BS), light from one path was imaged onto a Thorlabs DCC1545M CMOS camera using (L3) to allow for sample alignment, and the light in the other path was coupled into a multimode fiber (ThorLabs M14L10) to an OceanOptics HR2000+ES visible light spectrometer using L1.

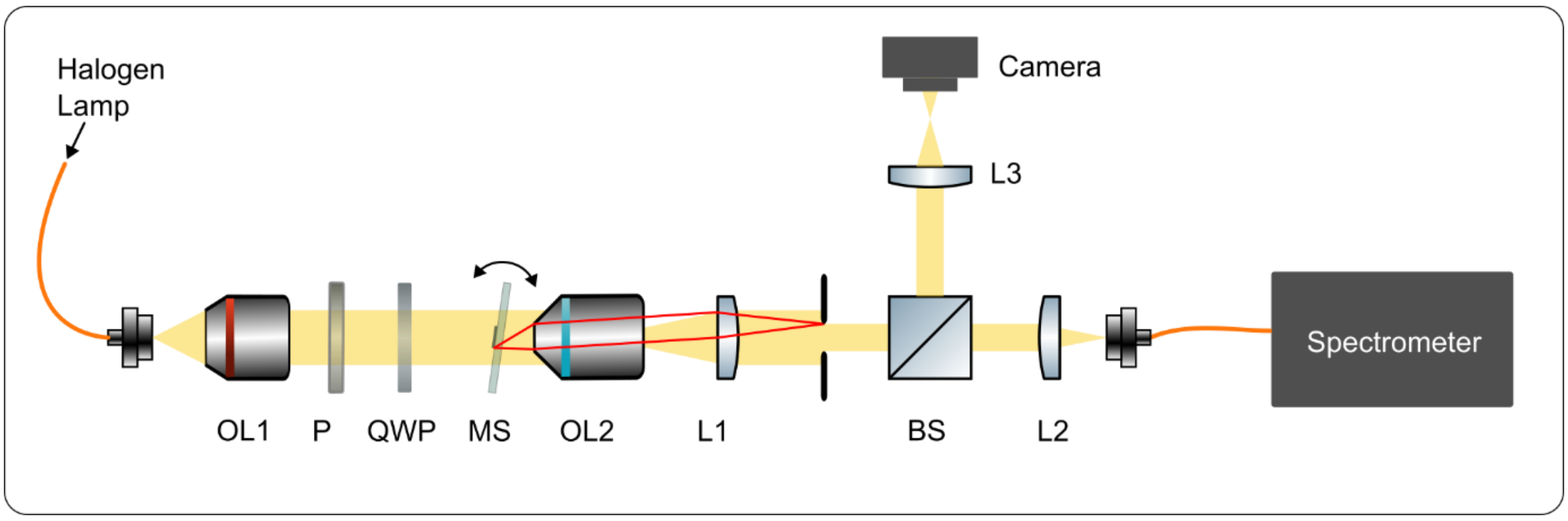

Supplementary Figure 5: Diagram of the spectroscopy setup.

Supplementary Figure 6 shows the diagram of the back focal plane imaging setup. The light was coupled through a single-mode fiber and collimated using an Olympus 4x 0.1NA objective (OL1). A polarizer (P, Thorlabs LPVIS050-MP2) was placed to set the incident polarization to be linearly polarized. The collimated beam illuminates the back aperture of an objective (OL2, Olympus Plan 20x 0.4NA) to create a focal spot on the metasurface. After passing through the metasurface, the light is then collected by an imaging objective lens (OL3, Olympus Plan 20x 0.4NA). The back focal plane of the imaging objective was then relayed to a polarization-sensitive camera (Thorlabs CS505MUP1) using a series of lenses (L2, L3 and L4). L2 is a biconvex 125 mm lens (Thorlabs LB1106-B), L3 is a 100 mm plano-convex lens (Thorlabs LA1509-A), and L4 is a 75 mm plano-convex lens (Thorlabs LA1608-A). A quarter waveplate (QWP, Thorlabs AHWP10M-580) is used such that circular polarizations can be distinguished by the polarization-sensitive camera instead of linear polarizations. The aperture seen in the camera corresponds to the numerical aperture of the imaging objective, given that the cone of light from the condenser has a numerical aperture greater than or equal to the imaging objective numerical aperture. This was used to correlate the pixel positions of captured images with incident directions, with normal incidence being at the center of the aperture. By sweeping through the illumination wavelength in small steps, we observed the evolution of the angular response to ascertain the wavelengths where the desired transfer functions occur.

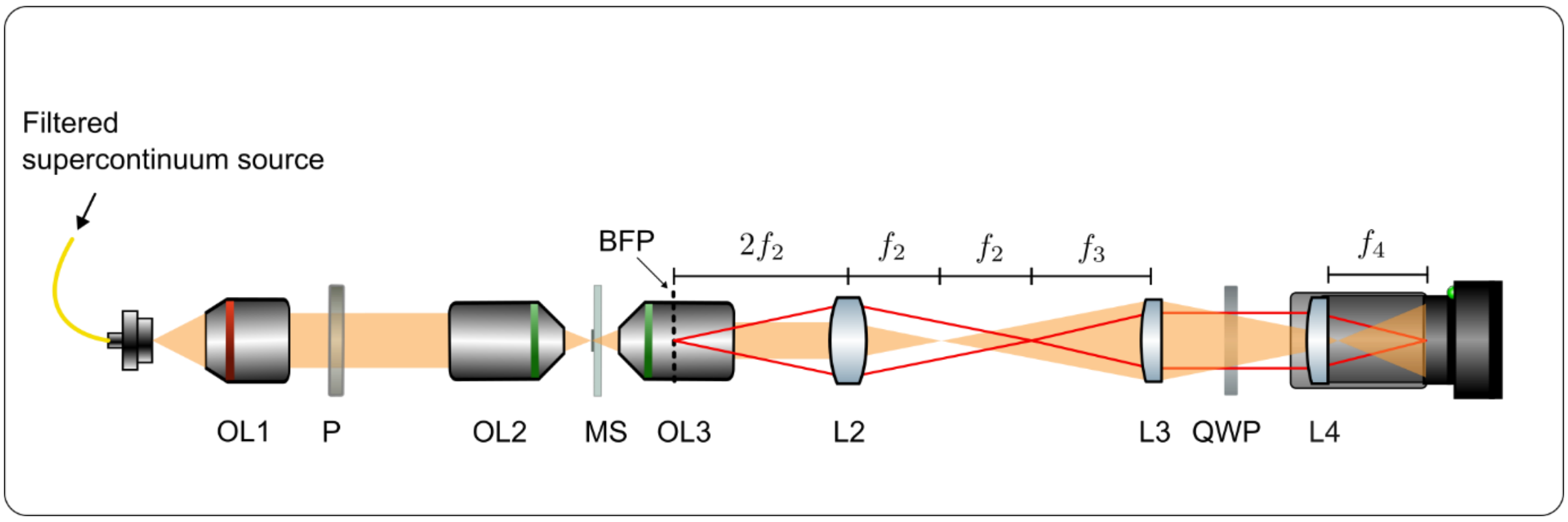

Supplementary Figure 6: Diagram of the back focal plane imaging setup.

Supplementary Figure 7 shows the diagram of the setup used to demonstrate phase imaging. The beam from the output collimator of the fiber-coupled filtered supercontinuum laser (NKT Photonics SuperK Compact with a SuperK Select acousto-optic tunable filter) was expanded using OL1 (Olympus Plan 20x 0.4NA objective) and L1 (Thorlabs LA1986-B) to illuminate the active area of the SLM (Holoeye Pluto 2 phase-only spatial light modulator). P is a linear polarizer (Thorlabs LPVIS050-MP2) used to set the polarization to horizontal polarization. The SLM was illuminated with horizontally polarized light, such that pure phase modulations are achieved without altering the output polarization state. The SLM was mounted on a two-axis rotation stage to control the tip and tilt angle for adjustment of the incident angle of the beam onto the metasurface downstream of the setup. The second lens system consisting of L2 (Thorlabs LA1433-B) and OL2 (Olympus Plan 20x 0.4NA objective) then demagnifies the field reflected from the SLM onto the metasurface. This image then acts as the object. After passing through the metasurface, OL3 (Nikon TU Plan ELWD 20x 0.4NA objective) collects the processed image and L3 (Thorlabs LA1433-A) images. A quarter waveplate (QWP) (Thorlabs AHWP10M-580) is placed before L3 with its fast axis oriented at $45°$ with respect to the x-axis such that the circular polarization components of the light are converted into horizontal and linear polarizations to be analyzed by a polarization-sensitive camera.

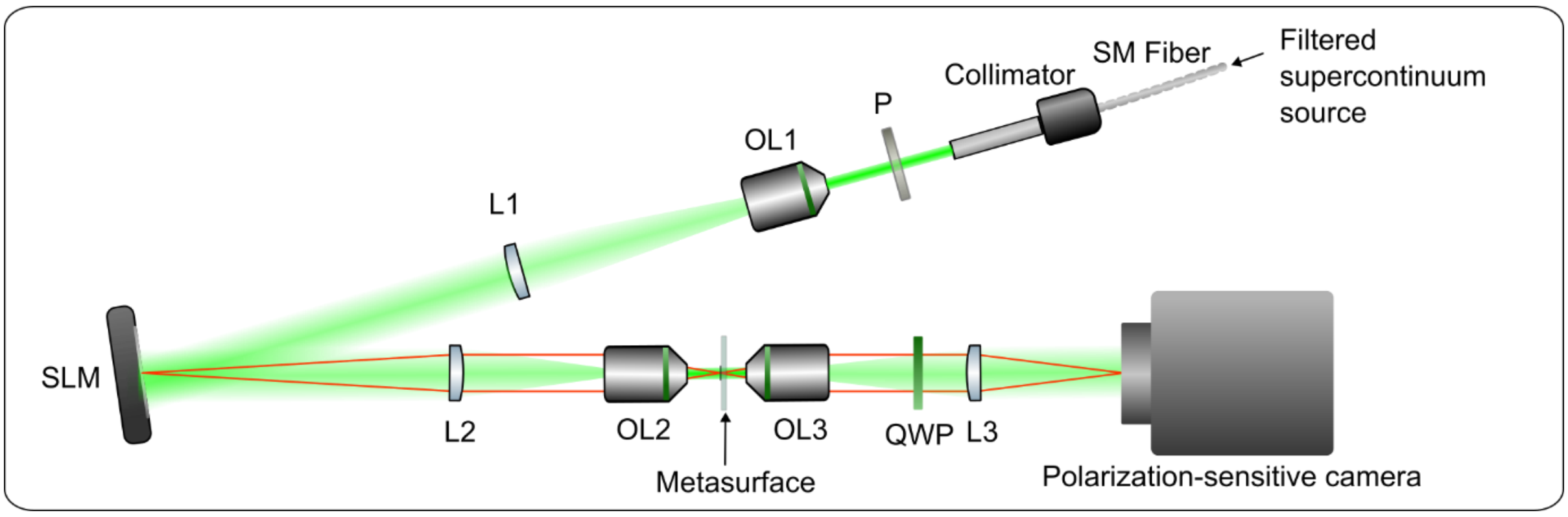

Supplementary Figure 7: Diagram of the phase imaging testbed.

References

1. Song, C., He, J. & Yuan, G. Generic full-vector angular spectrum method for calculating diffraction of arbitrary electromagnetic fields. *J. Phys. Photonics* **7**, 045021 (2025).
